# The role of emergency medical teams in Eswatini during the COVID-19 pandemic

**DOI:** 10.11604/pamj.supp.2022.41.2.32546

**Published:** 2022-05-02

**Authors:** Ngoy Nsenga, Didier Bompangue, Boniface Oyugi, Cornelia Atsyor, Nomthandazo Lukhele, Flavio Salio, Timothy Das, Camila Lajolo, David Anderson, Alison Mee, Andy Kent, Velephi Nhlengetfwa Okello, Ishata Nannie Conteh, Joseph Chukwudi Okeibunor, Zabulon Yoti, Abdou Salam Gueye

**Affiliations:** 1 World Health Organisation, Regional Office for Africa, Emergency Preparedness and Response Programme, Brazzaville, Congo,; 2 WHO Country Office Eswatini, Kingdom of Eswatini,; 3 World Health Organisation, Headquarters, WPE/CRS/HCR, Geneva, Switzerland,; 4 UK-Med Headquarters, United Kingdom,; 5 UK-Med Eswatini,; 6 Eswatini Ministry of Health

**Keywords:** COVID-19, Emergency Medical Team, Eswatini, WHO

## Abstract

The paper documents experiences and lesson learned in responding to COVID-19 pandemic in Eswatini with the support of the Emergency Medical Teams. WHO databases, operation reports and hospitalization records were reviewed. The WHO Emergency Medical Teams built the capacity of the local response teams in Eswatini. The conclusion is that following the intervention of the WHO Emergency Medical Teams, Eswatini is better prepared to respond to the ongoing COVID-19 pandemic and future outbreaks.

## Introduction

After the first wave of COVID-19 from June until the 30th of September 2020, the confirmed cases were 5,462 in Eswatini. However, in December 2020, the country experienced a huge surge in cases (6,419) and an alarming case fatality rate (1.9%). With the gaps in the healthcare systems, clinical management challenges, and patient contributing factors, the Eswatini Ministry of Health (MoH) was concerned that the already highly stretched teams would struggle to cope with a further rise in case numbers. Therefore, the MoH raised a request for assistance (RFA) from an Emergency Medical Team (EMT), which was shared with the World Health Organisation (WHO) Country Office (WCO).

*The Global Emergency Medical Team (EMT) Initiative was launched in 2014, following the Haiti earthquake and the publication of the Classification and minimal Standards for Foreign Medical Teams (GHC, 2013), recognising that « good intentions » are not enough to provide quality and life-saving interventions to people affected to emergencies. See overview in* ***Annex 1*** *Box 1 below*.

The RFA reached the EMT-secretariat in Geneva through the EMT-Regional Focal Point (RFP) in the WHO Regional Office for Africa (AFRO). The EMT´s focus was to support the care for the most serious and critically ill patients with staff and training, biomedical expertise to assist repairing, maintaining, and using critical care equipment, infection prevention and control and risk communications support. Following the request, the UK Emergency Medical Team (UK EMT) deployed 11 clinical and operational specialists in conjunction with their accountable grant partner, emergency health non-governmental organisation (NGO) UK Med.

The team worked in two treatment centres (Mavuso Treatment Centre and The Raleigh Fitkin Memorial Hospital) to build capacity and give a longer-lasting impact. UK-Med via the UK EMT provided 11 suitably trained and experienced staff who provided training and on-the-job supervision and clinical care working alongside national health staff. Additionally, they supported the local colleagues in understanding the need for preventative maintenance, repair and education for medical equipment, particularly those used for oxygen therapy. At the end of the assignment, the national HCW are now better prepared to respond to COVID-19 cases through early identification, containment, management of patients, improved clinical capability and health facilities optimisation.

This paper the contributions of the EMT to COVID-19 response in Eswatini, as documented by the country team. It also documents the country experiences and lesson learned in both building the capacity of the HCW and preparedness to respond to the ongoing COVID-19 pandemic and any possible future outbreaks.

### Eswatini: young, landlocked and strained

The Kingdom of Eswatini (known as Swaziland until 2018) is a small, landlocked country in Southern Africa that borders Mozambique to its northeast and South Africa to its south, west and north. It is divided into four regions (Hhohho, Lubombo, Manzini and Shiselweni). The country gained its independence from the British on September 6, 1968, and had a population of 1.14 million as of 2018 (projected from a population census of the year 2007), of which 59% are estimated to live below the international poverty line [1]. Eswatini has an average life expectancy at birth of 59.4 years (compared the Sub-Saharan Africa (SSA) average of 61.2 years); a Gross National Income (GNI) per capita of 8,070 US$ and 23.8% of the population is living in urban regions (compared the SSA average of 40.2%) [2]. The Human Development Index (HDI) is 0.608 (compared to the SSA average of 0.541) with a Global Health Security Index (GHSI) of 31.1 [3,4].

The country has 2.1 hospital beds per 1,000 population available and the health workforce is low, with 3.3 physicians (2016) and 41.5 nurses and midwives (2019) per 10.000 population [5-7]. Eswatini struggles with the highest Human Immunodeficiency Virus (HIV) prevalence worldwide (27,1 % of 15+ year-olds), causing major financial strain and economic instability [8,9]. Nonetheless, there has been a significant decline of acquired immune deficiency syndrome (AIDS)-related deaths and new HIV infections since 2010 (49% and 66% respectively) due to the wide availability of antiretroviral treatment (ART) treatment (>95% of diagnosed patients) [8]. Despite a decrease in the incidence (per 100,000 people) of tuberculosis by over 70% since 2010, still, 363 new cases per 100.000 population were detected in 2019, with 8.6% of new cases showing multidrug resistance, posing another ongoing epidemic in the country [10,11].

## Rationale for support requested

### The COVID-19 impact on Eswatini

**Figure 1:**
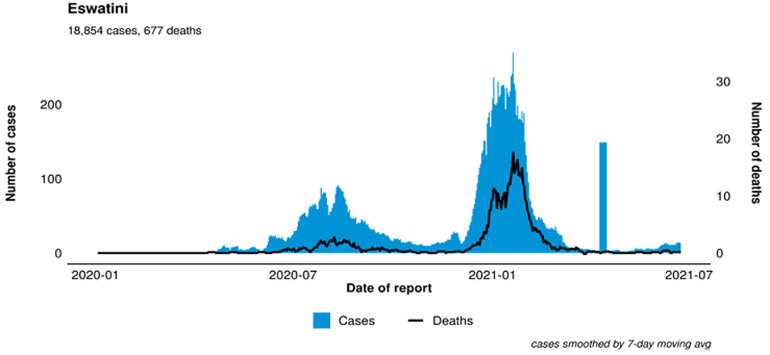
epidemiological curve, number of new COVID-19 cases diagnosed and associated deaths per week in Eswatini

Following the importation of the first COVID-19 case into Africa on February 14, 2020, in Egypt, and in the WHO AFRO Region on February 25 2020, Eswatini counted 18,854 confirmed cases of COVID-19 with 677 associated deaths as of June 23, 2021 [12]. Eswatini experienced a first wave of the virus between early June and the end of September, which affected all four country regions. From early October, case numbers had been low. Eswatini experienced a huge surge in coronavirus cases and an alarmingly high case fatality rate (Figure 1). Up to December 26, 2020, Eswatini had reported 8170 confirmed cases and 163 COVID-19 related deaths (Case Fatality Rate 1.88%). Within just one month, by January 27, 2021, this had risen to 517 deaths, and 14.831 confirmed cases (CFR 3.48%) [12]. The deaths included high-level persons, including political, religious and traditional leaders and other professional experts across sectors. Eswatini put 11 COVID-19 treatment centres in place (nine public and two private) with a total capacity of 437 beds, including 29 ICU beds. Eswatini had 37 beds at the end of the first wave (September 2020), and by April 2021, there were 102 (49 HDU beds and 53 ICU beds). The number of assigned COVID-19 beds was increased to 488 and later to 618 at the peak of the second wave, and currently (as of July 2021), there are 530 beds nationwide for COVID-19 patients and 53 critical care beds. The number of beds was increased based on the needs. At the height of the second pandemic wave, the country´s critical care beds capacity of 53 were full. It was impossible to expand Eswatini's ICU capacity due to healthcare workers (HCWs) and oxygen supply shortages.

### A Healthcare system struggling to cope

Given the COVID-19 experience in Eswatini, the gaps in the health systems were highlighted through a consultative forum and summarised as patient-related factors, clinical management-related factors, and health system challenges (Table 1). This assessment was done based on the WHO health systems framework that elucidates the overall performance of a health system to achieve its main goals (such as promoting good health outcomes, responding to population´s non-medical needs, financial protection against catastrophic health expenditures, and improving the efficiency of healthcare services) [13]. The health systems challenges fall mostly into the overwhelming building blocks of service delivery, health workforce, and access to essential medicines as summarised in Table 1 [14].

**Table 1:** gaps identified leading to increased mortality amongst COVID-19 patients in Eswatini in December 2020 - January 2021

Patient-related Factors	Clinical management related Factors	Health System Challenges
Delayed in seeking care Reluctance to initiate therapy. Comorbidities	Rapid weaning of oxygen therapy Infrequent monitoring of critically ill patients Lack of adequate tests such as D-dimers, blood cultures, arterial blood gasses.	Critical care support lacking (HR, training, equipment, drugs) Lack of transport to x-rays / lack of x-rays within the facility Lack of adequate nursing personnel to care for critically ill patients. 2 to 3 nurses catering for 30 to 35 severely ill patients Six fold increasing oxygen demands and lack of skills on oxygen escalation therapy. Lack of isolation beds in the peripheral health facilities[1] Lack of ICU beds Sedation/ICU med stock-outs

Given the challenges, medical teams in less well-equipped treatment centres saw country death rates increasing because all the hospitals were full and had nowhere to transfer their patients to. At the same time, neighbouring South Africa, Eswatini´s main supplier of oxygen, was also experiencing a surge in case numbers. As South Africa closed its borders, Eswatini´s oxygen supply was shut down. At the peak of the surge, Eswatini only had 52% of the oxygen needed for ICU patient care resulting in medical staff in Eswatini rationing oxygen for their patients. The system was not strongly resilient as other building blocks of health systems, including information systems, financing, and leadership/governance, were equally strained, thus leading to a further increase in COVID-19 related mortality [15] (**Annex 1**, Box 1).

### Request for assistance

In the structure of the incident management support team (IMST) of COVID-19, the incident manager (IM) oversees and coordinate the regional response to the pandemic. Under the IMST, one of the pillars is the health operations and technical expertise (HOTE) under which the EMT sub pillar fall. The EMT sub pillar has a focal lead who works closely with the different WHO country offices (WCO), the IM, and the MoH in the countries to support meeting the countries requests for I-EMTs. Also, the EMT focal point works closely/ collaborates with the case management teams at the WCOs. Given the pressure placed upon the local medical teams by the second surge in the pandemic, the Eswatini MoH were concerned the already highly stretched teams would struggle to cope with a further rise in case numbers. On January 1, The WHO AFRO COVID-19 Incident Manager received the following message from the WCO:

*We yesterday saw an all-time high mortality of 21 cases! The main issue is the overwhelming of the health system in the country especially for critical care. Apart from the discussions and requests mentioned yesterday, we will need surge capacity in critical care. We will immediately need at least 8 doctors and 8 Nurses with capacity to provide critical care and train and monitor Health staff in order to quickly expand capacity for critical care. We need 4 biomedical technicians to be able to install and ensure needed equipment for critical care like oxygen extractors and ensure they are well functioning. These cadres will be immediately deployed to the regions to provide onsite support training and mentoring to the treatment facilities in the 4 regions. Your urgent response will be appreciated.” -* ***Dr Cornelia Atsyor, WHO Representative - WHO Country Office Eswatini***.

The same day, the WHO AFRO EMT focal point shared the guidance for making an EMT request (**Annex 1**, Box 2) with the WCO. On January 2, the MoH signed the request for assistance and shared it with the WCO on January 3. On January 7, the request for assistance from an EMT reached the EMT-secretariat in Geneva through the regional focal point (RFP) in the WHO Regional Office for Africa (AFRO). The EMT secretariat worked with the Regional Office, the WCO and the MoH in Eswatini to clarify the request, which was finally shared with the wide EMT network on January 13. In the Eswatini MoH request for assistance to the WHO EMT Secretariat, the areas of need identified aimed to fulfil the gaps leading to increased mortality, as per Table 1. It included specialist ICU support for staff caring for the most serious and critically ill patients, biomedical expertise to assist the fixing, maintenance and use of critical care equipment, infection prevention and control and risk communications support.

## WHO EMT response framework

Alerts can be sent to the global EMT secretariat by any member state authority or a WHO regional or country entity. The more detailed the alert is, the better tailored the event notification sent by the EMT secretariat to the teams will be. Details can include specific clinical profiles, equipment needs, expectations regarding self-sufficiency, typology of the team requested etc. The EMT secretariat will notify the teams registered in the EMT network and collate the received expressions of interest in a list with the teams and modalities of deployment. A specific form was developed for teams to use when expressing their interest. This list will be shared with the requester, the RFP, and the WHO Country Office (WCO). RFP and WCO review the offered teams against the alert they raised initially and select the most suitable offers. Details of the mission are defined between WHO and/or the member state and the selected teams. After formal acceptance by the health authority of the member state concerned, through the WCO, the selected team is formally notified by WHO and mobilised.

On January 20, the UK EMT came forward with an offer fitting the support requested, then proposed to and accepted by the Eswatini MoH. The UK Emergency Medical Team deployed 11 clinical and operational specialists in conjunction with their accountable grant partner, NGO UK Med. The team was made up of a logistician, biomedical engineer, two critical care doctors, two critical care nurses, two infection and prevention and control nurses, a specialist in risk communications, an emergency department nurse as medical coordinator and a consultant orthopaedic surgeon to lead the team. Four team members were British, and the other team members were from Nigeria, Kenya, the Democratic Republic of Congo, Rwanda and Zambia. The team had between them worked on Ebola, diphtheria, measles and COVID-19 outbreaks around the world (**Annex 1**, Box 2).

### Implementation of EMT-support

On January 30, on arrival, and in coordination with the WCO and the MoH, the UK EMT were asked to support two treatment centres in Manzini. Manzini region had the highest cumulative deaths in the country (40%), with the second-highest cumulative confirmed cases (6,183) [16]:

1) The Mavuso Treatment Centre (an exhibition centre initially set up in June 2020 to receive isolation and minor cases) has 143 beds but no hospital infrastructure. During the recent surge in cases, it has had to manage moderate cases (adolescent or adult with clinical signs of pneumonia (fever, cough, dyspnoea, fast breathing) but no signs of severe pneumonia, including SpO2 = 90% on room air) and severe cases (adolescent or adult with clinical signs of pneumonia (fever, cough, dyspnoea, fast breathing) plus one of the following: respiratory rate > 30 breaths/min; severe respiratory distress; or SpO2 < 90% on room air) with inadequately trained staff and equipment [17]. The facility was well-equipped to manage moderate cases; however, there were no step-up beds to support the care of severe cases, which therefore necessitated transfer to an appropriate ICU bed within Eswatini.

2) The Raleigh Fitkin Memorial Hospital (RFM) is a well-established tertiary referral hospital with a small (but overwhelmed) ICU. The hospital has 350 Beds and 8 ICU beds. The hospital provides services to around 500 patients in the outpatient department daily. In addition, patients have access to HIV/AIDS mitigation programs, including PMTCT (Prevention of Mother to Child Transmission), VCT (Voluntary Counselling and Testing), ART (Anti-Retroviral Therapy) and a supplementary feeding program sponsored by the World Food Program (WFP). While the hospital is well managed, it had insufficient capacity to deal with the significantly high volume of COVID-19 cases that presented. Therefore, it referred moderate cases to Mavuso Treatment Centre, and severe cases were transferred to the most appropriate ICU beds within Eswatini.

Given that the patient admissions for COVID-19 started to drop (plausibly because of the containment measures by the government) and with fewer patients to treat, the team repositioned their support to prepare the already exhausted staff for a predicted third wave of the virus. Initial assessments of the two facilities by the UK EMT, done and adapted WHO hospital readiness checklist, resulted in the development and implementation of a plan for areas of training, support and supervision, which had been highlighted, both by the team's initial assessment and by the teams working in the facilities [18]. Capacity-building activities were provided through knowledge transfer and on the job training, clinical supervision and working alongside the facilities HCW. Identified areas of support were: 1) Optimising facilities to create clinically appropriate patient and staff flows, putting in place a triage and admission process for COVID-19 and training national staff in how all these new systems work. 2) Supporting the appropriate identification and screening of patients. 3) Ensuring isolation and clinical care for all cases, including severe and critical patients in need of enhanced levels of care. According to context needs and resource availability, the adaptation of clinical guidelines and the development of standard operating procedures for the identified facilities. 4) Clinical training, on-the job supervision, and working alongside health staff dealing with COVID-19 cases of all levels. 5) Implementation of appropriate infection prevention and control (IPC) measures and delivery of IPC training to national staff, specifically for COVID-19. 6) Biomedical support to the sites for repair and maintenance of essential equipment. 7) Support to the regional plan for increasing oxygen supply, moving towards self-sufficiency in this area. 8) Risk Communications and Community Engagement (RCCE) activities. 9) Supporting the surveillance system within the facilities or areas identified through the optimisation of existing data collection systems.

To quantify this clinical impact, feedback forms for all training delivered were introduced and documented. Observational findings demonstrated significant improvements in Personal Protective Equipment (PPE)-usage (esp. donning & doffing), basic infection, prevention and control measures, oxygen administration, escalation of deteriorating patients and earlier transfer of severe cases to appropriate facilities. The teams at both sites were incredibly open to training and welcomed the opportunity to enhance their skills with additional learning and mentorship. On request of the MoH, the team´s Biomedical engineer´s role was expanded to include not only delivering training in the maintenance and use of critical care equipment in the two treatment centres but to look at the wider issue of developing a plan for creating sustainable oxygen supplies throughout the country. The team´s RCCE - specialist Dr Ngoni who was initially tasked to support the RCCE-activities in the local Manzini region, was asked to advise regionally and nationally on improving the RCCE-strategy.

## Immediate outcome

**Figure 2:**
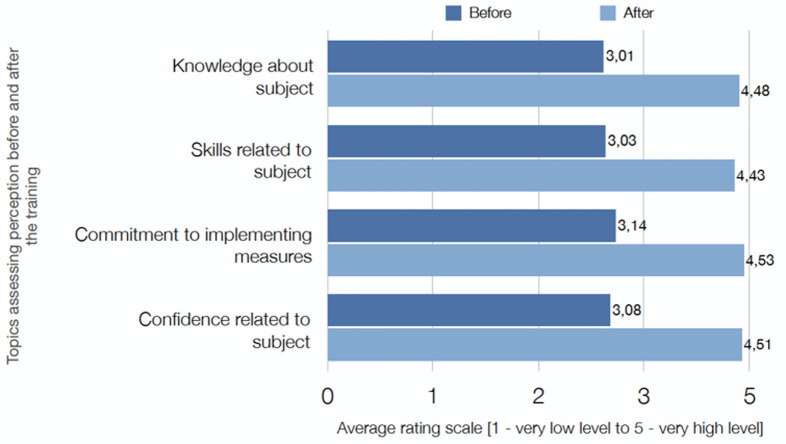
chart displaying the average rating of 179 responses. In all four areas accessed, participants positively rate the training as having improved their knowledge, commitment, skills, and confidence

**Figure 3:**
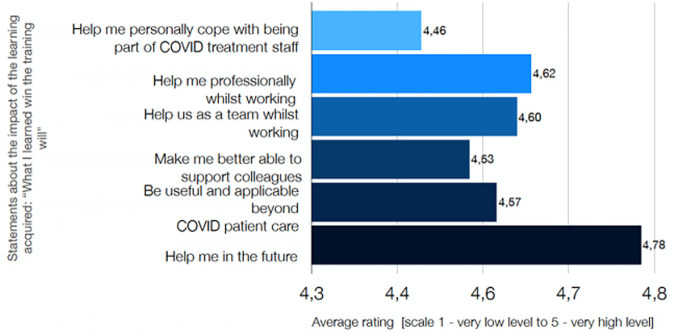
chart displaying the average rating on a 1-5 scale of 179 responses. Respondents agreed or strongly agreed with the statements shown. (Note: the x-axis has been truncated)

The UK EMT measured the potential effects of the training and support provided by asking the participants to rate their perceptions of knowledge, skills, commitment to implement and confidence. Participants´ responses were measured on a 5-point Likert Scale and averaged for reporting purposes. The overall rating of training was 4.5/5.0, and all measures showed a marked increase, as demonstrated by Figure 2 and Figure 3 below on perceptions before and after training. More importantly, the training, supervision and mentorship had a significant impact upon patient care, particularly for those patients with Moderate/Severe disease. There was earlier identification and escalation of care for patients when they began to deteriorate, with appropriate oxygen therapy and CPAP. An excellent example of the team's impact is laid out in the below extract from a real-life experience at RFM hospital (**Annex 1**, Box 3). The patient story shows, be it on a single sample level, there is a clear result of implementing training and locally adapted protocols for patient outcomes. The patient deterioration was identified early, care was adapted and escalated according to protocol, and referral was timely initiated.

**Figure 4:**
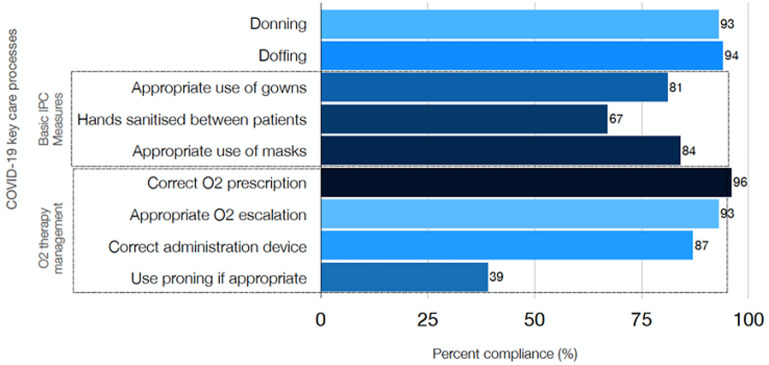
compliance with COVID-19 selected key processes at two facilities in Eswatini. Data was collected during ward rounds at Mavuso and RFM health facilities using a structured data collection form specially designed for that purpose over 4 days in March and included 46 patient days: median patients per round 4,5 [3-11]

During the last week of clinical activities, the UK EMT observed the compliance with selected, previously trained clinical processes using a structured checklist during ward rounds on four different days. There was a marked improvement shown compared to the start of the support. The area with the lowest compliance, patient proning, was partly because this was an entirely new concept to the staff and had only been introduced one week before the audit was carried out (Figure 4). Appropriate use of PPE continued to be a challenge, with the staff having major concerns around the quality of the KN95 masks used during aerosol-generating procedures. This invariably led to 'double' masking with the use of a surgical mask and KN95.

## Lessons learned in expanding and sustaining the gains

During the last weeks of the deployment, the MoH, WCO and UK EMT jointly organised a Training of Trainers in key areas to support the ongoing development of the care system in Eswatini. A one-week program was put in place focusing on the appropriate IPC, PPE, Basic Life Support (BLS) and Escalation of care. This training was attended by staff from all MoH care facilities and the main private sector hospitals and followed by a week of placement support and 1: 1 supervision to ensure that the learning could be embedded. Significant investments were made in ensuring that the countrywide SOPs and Case Management tools were updated and implemented across all COVID-19 receiving facilities in Eswatini.

Being one of the gaps that the MoH identified and requested for support, the MoH and WHO is considering funding a four-week biomedical engineer course to ensure that the technicians and engineers have the skills, abilities and tools to continue with the work initiated as part of the deployment. Incidentally, in all the AFRO countries, COVID-19 has shown that biomedical engineers are a significant gap in meeting emergency needs. On another side, this deployment was an opportunity for the MoH to discover the added value of the EMT initiative. The MoH and WHO agreed to start working on the development of an Eswatini national EMT.

## Conclusion

The MoH and WHO had a clear vision of what was required from the UK EMT when the request went out to support the people of Eswatini. However, with the declining number of cases (plausibly because of the containment measures by the government), the requirement to work within ICUs to deliver direct care was decreased. This meant an increased opportunity for the UK EMT to work with the national staff to build capacity and give a longer-lasting impact with support across all the key outbreak pillars. The UK EMT has provided 11 suitably trained and experienced staff, who provided training on the job-supervision and clinical care working alongside national health staff in the pre-identified health facilities (Mavuso Treatment Centre and Raleigh Fitkin Memorial Hospital), targeting COVID-19 cases as per set case definitions defined by the WHO and in line with the Eswatini MoH, ensuring health activities were technically sound and of high quality.

Biomedical engineering played a key part in the response and was invaluable in supporting local staff in understanding the need for preventative maintenance, repair, and education for medical equipment. And, those items helped to support good and appropriate use of oxygen therapy. There is also a need for additional training and support for the Eswatini Biomedical Engineers to ensure they have the most up-to-date knowledge, skills, and equipment to deliver high-quality support. National HCW are now better prepared to respond to COVID-19 cases through early identification, containment, and management of patients, improved clinical capability and health facilities optimisation. These results represent the average assessment of 19 trainings encompassing different COVID-19 related topics conducted by the EMT. The total number of assessments was 179, and the median number of assessments per training was 10 (CI: 2-17) (some people attended more than one training). Most staff trained were HCW from the facilities, but some were anonymous. Examples of topics included COVID-19 case management, critical patient monitoring, IPC, RCCE, basic life support, bio-med patient monitoring, ARI, and emergency trolley.

Supporting Eswatini MoH to implement its own National EMT will be one of the key next steps to help to strengthen the country´s capacities to timely and effectively respond to emergencies and outbreaks at national and sub-national levels when they occur. In collaboration with the EMT AFRO, the MoH is committed to achieving this objective and plan to implement the national EMT. The implementation will help achieve and maintain the EMT Minimum Standards, combined with the highest possible level of operational readiness. Moreover, gaps in Eswatini health systems brutally highlighted by the COVID-19 pandemic need to be rapidly addressed by building and strengthening health systems that can withstand the stress and adapt to the demands generated by emergencies of that scale and magnitude whilst maintaining the delivery of essential health services throughout.

## Annex

**Annex 1****:** overview of the UK EMT: initiatives, organization and testimony
